# Factors that influence smokers’ and ex-smokers’ use of IQOS: a qualitative study of IQOS users and ex-users in the UK

**DOI:** 10.1136/tobaccocontrol-2019-055306

**Published:** 2020-01-15

**Authors:** Charlotte N E Tompkins, Annabel Burnley, Ann McNeill, Sara C Hitchman

**Affiliations:** 1 Department of Addictions, Institute of Psychiatry, Psychology & Neuroscience, King’s College London, London, UK; 2 SPECTRUM Consortium, UK – Shaping Public Health Policies To Reduce Inequalities and Harm, UK

**Keywords:** tobacco industry, harm reduction, non-cigarette tobacco products

## Abstract

**Background:**

One of the most widely available heated tobacco products is IQOS by Philip Morris International. However, there is a lack of independent research exploring IQOS initiation and subsequent use among smokers and ex-smokers.

**Aims:**

To (1) explore the reasons why smokers and ex-smokers use and continue/discontinue IQOS and (2) consider implications for future research and policy.

**Participants:**

Adult (18+) current (n=22) and ex-users (n=8) of IQOS who either currently smoked or quit smoking in the last 2 years.

**Methods:**

Qualitative interview study in London, UK.

**Results:**

Six main factors influenced initiation and use of IQOS: (1) Health—wanting to reduce/quit smoking and perceptions of reduced harm (while understanding IQOS was not risk-free). Branded packaging, absence of pictorial warnings and physical health improvements conveyed reduced harm. (2) Financial—including high start-up costs, but cheaper ongoing costs than smoking. (3) Physical—mixed views on enjoyment and satisfaction. Sensory experiences influenced use including discreetness, cleanliness, reduced smell and tactile similarities relative to combustible cigarettes. (4) Practical—issues of accessibility, shortcomings with maintenance/operation limited ongoing use, whereas use in smoke-free places increased use. (5) Psychological—similarities in rituals and routines, although new practices developed to charge and clean; some liked trailblazing new technology. (6) Social—improved social interactions from using IQOS instead of smoking, but with more limited shared social experiences for some.

**Conclusion:**

For some, IQOS facilitated smoking substitution. Factors such as packaging, labelling, risk communication, price and smoke-free policies appear to influence initiation and use.

## Introduction

**Figure 1:**
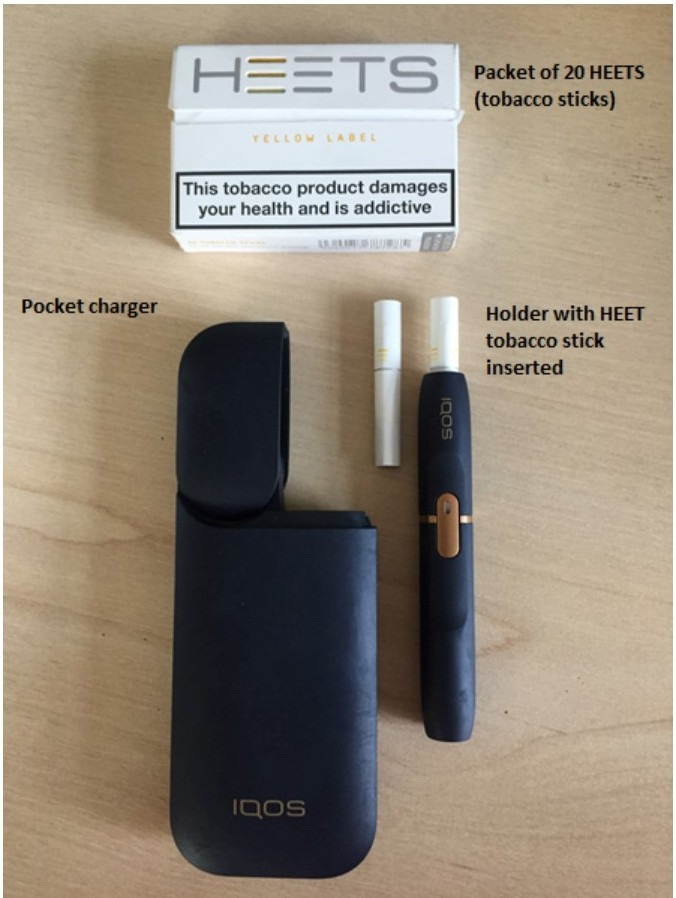
IQOS 2.4 Plus and HEETS tobacco sticks.

The IQOS battery-powered heated tobacco product (HTP) by Philip Morris International (PMI) was launched in 2014^1 2^ (figure 1). PMI claims that IQOS heats rather burns tobacco sticks (branded HEETS), thus avoiding burning and emitting “95% lower levels of harmful chemicals compared with cigarettes”.^3 4^ Available in over 40 countries, PMI estimate that 6.6 million smokers have quit smoking and switched to IQOS.^5^

**Figure 2:**
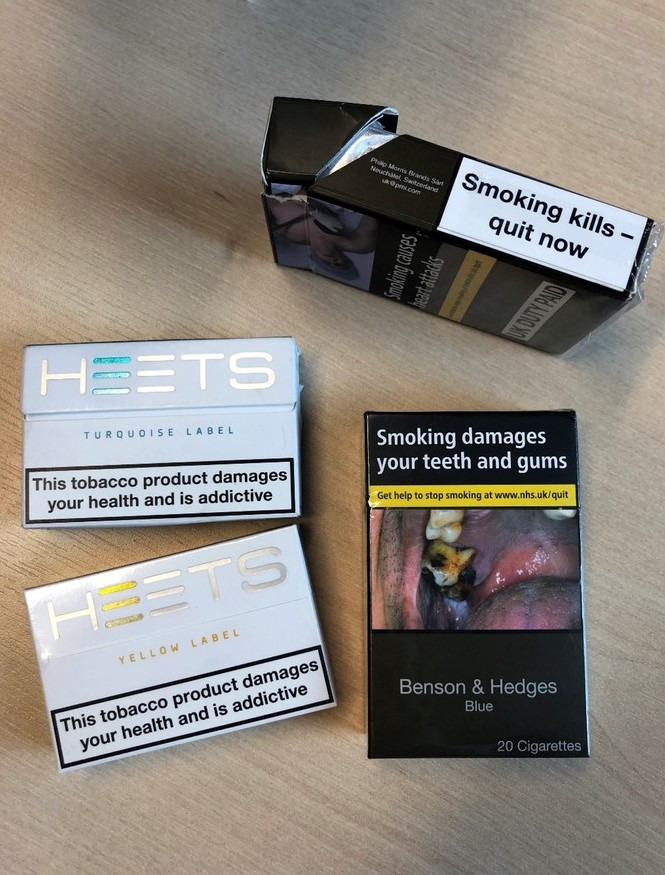
HEETS tobacco sticks and combustible cigarette packaging in the UK.

Since the 2016 UK launch of IQOS, sale locations have expanded beyond official IQOS stores to other retailers.^6 7^ Prevalence of IQOS use in the UK is uncertain. One study estimated overall HTP use at 0.82%; however, the measure used may have been problematic.^8^ IQOS advertising appears in the UK, but HEETS advertising does not. IQOS retails between £59 and £89; HEETS retailed at £8 for a packet of 20 until July 2019 when PMI reduced the price to £5, less than half that of most combustible cigarettes.^9–11^ HEETS are not subject to standardised packaging and pictorial warnings, and only require a 30% black-and-white text warning (figure 2). There are no official policies on using IQOS in public.

In April 2019, the US Food and Drug Administration (FDA) authorised IQOS and ‘Heat Sticks’ for marketing in the USA.^12^ Research (mostly tobacco industry funded) suggests that, compared with combustible cigarettes, there are reduced toxins in the aerosol produced by IQOS; IQOS may reduce users’ exposure to some “harmful and potentially harmful constituents”; and some biomarkers of harm may be reduced when switching from smoking combustible cigarettes to IQOS.^13–20^ However, the public health impact of IQOS will depend on the degree to which use is less harmful than smoking, whether smokers switch completely to IQOS, and the extent of non-smoker uptake.^17 21^

Most research on consumer responses to IQOS has been conducted by PMI.^22–25^ Independent survey research found that IQOS is perceived as less harmful than smoking.^26–28^ Focus group studies in Switzerland and Japan found IQOS was more socially acceptable, cleaner and uptake was encouraged by ‘clean and chic’ marketing and less of a harsh feeling on the throat compared with cigarettes.^29^ However, the bulkiness of the device, charging/cleaning, cost and the odd smell discouraged use.^29^ Popular reasons for using IQOS identified in a Swiss study included complete smoking cessation, the reduced toxicity relative to smoking, the reduced smell and the improved taste.^28^ Most users found IQOS weaker on the throat than combustible cigarettes and reported improved physical health, but feared becoming dependent.^28^

Due to increased availability of IQOS, its use for harm reduction and its rapid uptake in some countries,^30^ it is crucial that we develop a better understanding of smokers’ perceptions and responses to IQOS. Thus, we conducted a qualitative study with IQOS users and ex-users in London to explore and understand the factors that encourage and discourage current and ex-smokers to initiate, continue, and discontinue IQOS use. We used interviews to explore individuals’ experiences in detail and to obtain a greater depth of data than could be generated from focus groups.

## Methods

Eligibility criteria were adults (18+) who (1) currently smoked *or* quit smoking in the last 2 years, (2) use *or* used IQOS at least weekly for at least 1 month, and (3) lived in the UK. We recruited online (research recruitment website, classifieds, social media) and via a university-wide email. Posters were also displayed in vape shops selling IQOS across London and we approached individuals using IQOS in public. After screening, eligible individuals were selected for interview based on their demographics and smoking/IQOS histories.

Between October 2018 and February 2019, CNET, a qualitative researcher and joint lead author, interviewed 30 people (28 face-to-face, 2 by telephone). A semistructured topic guide, informed by consultations with seven tobacco/nicotine experts and a panel of 12 current and ex-smokers, guided the interviews. It covered participants’ smoking history, motivations and experiences of using IQOS (including reasons for continued and discontinued use), the benefits and drawbacks of IQOS, and the perceived impacts of IQOS (overall and on their smoking). Participants were offered a £20 shopping voucher. Interviews lasted 36–102 min (mean=67).

Iterative categorisation, a rigorous and transparent approach, guided data coding and analyses.^31^ The authors read transcripts to familiarise themselves with the data, discussed the content, and developed a coding frame of deductive (based on areas included in the topic guide) and inductive (based on additional areas that arose during the interviews) thematic codes. The transcripts and the coding frame were imported into MaxQDA; CNET systematically coded each transcript and assigned all text to the relevant code/s. Then, AMcN, SCH and CNET jointly reviewed the coded data relating to why participants initiated, continued, and discontinued IQOS more inductively. They consolidated and re-organised the codes into health, financial, physical, practical, psychological and social factors. During analysis, the similarities and differences between participants were explored. Key findings are illustrated using pseudonymised verbatim quotations.

## Findings

### Sample

Participants included n=22 current and n=8 ex-IQOS users, all UK residents from a variety of nationalities (table 1). Many had smoked or used nicotine products for 20+ years; most had tried e-cigarettes (table 2 and online supplementary tables 1–3). All currently used or had used IQOS 2.4 Plus.

10.1136/tobaccocontrol-2019-055306.supp1 Supplementary data

**Table 1:** Demographic characteristics (n=30)

Demographic	Ex-IQOS use	Current IQOS use	Total
	(n=8)	(n=22)	(n=30)
Age (years)			
18–24	4	3	7
25–34	1	7	8
35–49	3	9	12
50–59	0	3	3
Total age range	18–48	22–59	18–59
Median	25	36.5	32.5
Mean	28.6	36.7	34.6
Gender			
Male	4	15	19
Female	4	7	11
Ethnicity			
White British	2	6	8
White Other*	5	12	17
Asian/Asian British†	0	3	3
Black/Black British‡	1	0	1
Arabic	0	1	1
Occupation			
Professional/qualified work	2	7	9
Managerial/senior administrator	2	7	9
Clerical/junior administrator	2	6	8
Sales/services	0	1	1
Semi-skilled/unskilled labour	1	0	1
Never worked	1	1	2
Current working status			
Full time (30+ hours)	2	18	20
Part-time	2	1	3
Currently unemployed	1	0	1
Part-time student, part-time employment	2	2	4
Full-time student	1	1	2

*These included 1 Australian, 3 Bulgarians, 1 German, 1 Irish, 3 Italians, 1 Lithuanian, 1 Northern Irish, 2 Portuguese, 1 Russian, 1 Swiss, 1 Turkish and 1 Ukrainian.

†These included 2 Indian British and 1 Sri Lankan British.

‡This participant was Black British.

**Table 2:** Overview of participants’ use of IQOS, combustible cigarettes and e-cigarettes (n=30)

	Ex-IQOS use (n=8)	Current IQOS use (n=22)	Total (n=30)
First tried IQOS			
1–3 months ago	0	3	3
4–6 months ago	0	3	3
7–12 months ago	3	8	11
More than 12 months ago	5	8	13
Length of IQOS use			
1–3 months	1	3	4
4–6 months	3	4	7
7–12 months	1	10	11
More than 12 months	3	5	8
Country/region first tried IQOS			
UK	6	15	21
Continental Europe*	2	5	7
Japan	0	1	1
South Africa	1	0	1
Cigarette smoking status			
Daily	4	3	7
Weekly	0	4	4
Monthly	1	3	4
Less than monthly	1	5	6
Not at all	2	7	9
E-cigarette use status			
Daily	1	1	2
Weekly	0	0	0
Monthly	1	0	1
Less than monthly	0	1	1
Ex-e-cigarette use**†**	5	19	24
Never e-cigarette use‡	1	1	2
Time using tobacco and nicotine products (years)			
1–5	4	5	9
6–10	0	5	5
11–20	2	1	3
More than 20	1	11	12
Total range (years)	1–22	2–40	1–40
Median (years)	14	17	14
Mean (years)	9.7	17.7	15.7

*These included Bulgaria, Italy, Lithuania, Switzerland and Ukraine.

†As defined by answering “How often do you currently use e-cigarettes?” as “Not at all” and answering “Have you ever previously used e-cigarettes?” as “Yes”.

‡As defined by answering “Have you ever previously used e-cigarettes?” as “No”.

§This includes use of other tobacco and nicotine products during this period (eg, shisha and cigars). One participant did not disclose this information in a specific enough way, so exists as missing data. N.B. This also excludes large periods of time (over 1 year) in which successful quit attempts were made (ie, these years were removed from the total time spent using tobacco/nicotine products).

Although we recruited smokers and ex-smokers, the interviews highlighted that smoking, IQOS and e-cigarette status was more complex. For example, some people identified as ex-smokers but when interviewed described recent situations where they smoked combustible cigarettes. Consequently, the quotations from people who said they no longer smoked at all (ex-smokers) are labelled as ‘former smoking’ to reflect that they have former experience of smoking but are not necessarily ex-smokers.

### Health factors

#### Smoking goal

Commonly, participants said that they experimented with IQOS because their goal was to reduce or stop smoking combustible cigarettes due to health risks. They thought that IQOS potentially provided a new and *‘healthier’* path away from cigarettes. Some explained this goal differently—with no intention of ‘*giving up*’ or ‘*quitting*’ altogether, they perceived IQOS offered an alternative way to carry on ‘smoking’. While these individuals started IQOS to continue smoking (using it instead of combustible cigarettes), they hoped that their health would benefit by replacing combustible cigarettes with HEETS.

*I thought well I don’t want to go on smoking so many cigarettes… this is not doing my health any good… I wanted to do something about it, and stopping didn’t seem to be a realistic option, so I wanted to try this (IQOS).* (Clive, 59, current IQOS user, less than monthly smoking)

Smoking goals also contributed to ongoing IQOS use. Although some reported that they had successfully used IQOS to stop smoking combustible cigarettes, few had quit smoking both combustible cigarettes and HEETS altogether, as they used HEETS in place of, or alongside combustible cigarettes.

#### Perception of harm

Participants repeatedly reported that they tried IQOS because they believed it was ‘*better*’, ‘*less harmful*’, ‘*less hazardous*’ or ‘*less damaging*’ for their health than combustible cigarettes. This view originated from the well-documented harms of combustible cigarettes, claims participants had read on PMI marketing material about IQOS, and the HEETS packets. Participants commented that HEETS packets were ‘*cleaner*’ than combustible cigarettes as they had ‘*less alarming*’ written health warnings and did not contain graphic pictorial warnings.

*I looked into IQOS and it did claim that it had… fewer harmful chemicals than a regular cigarette… That was part of the appeal. And I think that’s how it was marketed to me, that it was less harmful*. (Alison, 35, ex-IQOS user, former smoking)

Believing IQOS was less harmful than smoking combustible cigarettes motivated continued use, often prompting participants to use up to twice as many HEETS as combustible cigarettes, and discouraging quitting IQOS.

*Smoking cigarettes, I knew I was doing something really, really bad, and that I should… stop… whereas with this (IQOS), because I don’t know that it’s so bad for me, there really is no input or drive or motivation for me to think, ‘stop!’* (Francesca, 46, current IQOS user, former smoking)

At the same time, participants said that their mistrust of the tobacco industry and the ‘*lack*’ of independent evidence created uncertainty about the potential harms of IQOS. These issues did not discourage participants from trying IQOS but prompted them to seek more information and to consider their ongoing use. A few participants who found reports which contradicted claims of reduced harm debated discontinuing IQOS to return to combustible cigarettes.

*I read a recent systematic review saying that IQOS is as harmful as cigarettes! So I thought well I might as well smoke cigarettes and not the IQOS.* (Yasmina, 25, current IQOS user, weekly smoking)

Participants acknowledged that IQOS was unlikely to be risk-free because HEETS packets carried a warning and they had seen disclaimers on the PMI IQOS website and in IQOS stores. Furthermore, they perceived potential harms from ‘*inhaling something*’ into the lungs and from the tobacco, chemical and nicotine composition of HEETS. Nevertheless, they said that without detailed information about the ingredients and nicotine content of HEETS and in absence of longitudinal and independent research into IQOS, they tried to remain optimistic about the harms of IQOS compared with combustible cigarettes. While participants believed that they would re-evaluate their IQOS use if evidence uncovered health risks, some questioned whether knowing the risks would influence their use given that they had not quit smoking combustible cigarettes despite knowing the harms.

#### Physical health indicators

Participants often reported that their physical health improved since they started using IQOS—they felt ‘*fitter*’ and more energised, had fewer breathing difficulties, noticed increased cardiovascular ability and coughed less. Such improvements reinforced their perceptions that IQOS was less harmful than combustible cigarettes and encouraged continued use.

*I do find myself a bit less breathless, if I’m doing some exercise, or even walking up several flights of stairs, than I would have been… when I was smoking 10 cigarettes a day. Which… goes back to one of the reasons why I perceive it to be no more harmful, and if I’m honest probably slightly less harmful than cigarettes.* (Sanjay, 43, current IQOS user, less than monthly smoking)

Conversely, no participants reported direct experience of adverse health consequences resulting in second thoughts about using IQOS or discontinuation of IQOS use.

### Financial factors

#### Start-up and ongoing costs

Participants regularly expressed concerns that start-up costs of IQOS discouraged initiation. While free demos encouraged them to try IQOS, they felt that IQOS was ‘*expensive*’ and too costly compared with cigarettes or e-cigarettes to appeal to low-income smokers. To overcome financial barriers, some participants received IQOS as a gift, purchased IQOS secondhand, or took advantage of promotions or more affordable prices in mainland Europe. Nevertheless, participants with fewer financial constraints considered that the price of IQOS, although ‘*not cheap*’, was ‘*worth it*’ if it was ‘*better*’ than combustible cigarettes.

*I thought it was worth me spending the £80, or whatever it was for the IQOS and a few free packets (of HEETS), even if it turned out not to be something I stuck to.* (Raj, 43, current IQOS user, former smoking)

There were mixed views across participants on how the cost of IQOS influenced their continued use. A key driver for continuing to use IQOS was that HEETS were cheaper than combustible cigarettes, particularly when purchased in bulk via the PMI website or from countries with lower prices. Yet, participants who had previously smoked rolling tobacco or used e-cigarettes complained that HEETS were less affordable. As such, cost discouraged continued IQOS use for those with limited finances.

*A big factor (in stopping IQOS) was the cost… if I get it (HEETS) online… that will cost me a lot of money, and I have to be really careful with how I manage my money, because benefits (social security payments) only stretch so far.* (Maria, 29, ex-IQOS user, daily smoking)

### Physical factors

#### Enjoyment and satisfaction

Participants gave mixed accounts regarding how enjoyable and satisfying IQOS was. Reflecting on their first experiences, some commented that the similarity of the physical ‘*kick*’ of nicotine with smoking combustible cigarettes made IQOS enjoyable to use and easy to switch to. Others were ‘*underwhelmed*’ or ‘*disappointed*’ by what they described as a ‘*lighter*’ and ‘*less strong*’ physical feeling but found that they grew accustomed to this with continued use, and rarely discontinued using IQOS because of it.

Generally, current users reported that the inhalation from IQOS felt ‘*smoother*’, ‘*cleaner*’, ‘*less harsh*’ and ‘*lighter*’ in their throats than that of combustible cigarettes. They described how this encouraged them to use IQOS and provided a ‘*pleasant*’ and ‘*enjoyable*’ experience like smoking cigarettes but with fewer negative physical feelings in the throat.

*I immediately thought yeah, this is like smoking… I get the same reward… It’s very similar to smoking because of that famous kick that you get… because I just use it in exactly the same way as I used a cigarette.* (Francesca, 46, current IQOS user, former smoking)

Nevertheless, some remained uncertain about using IQOS. For example, they said that the experience was less satisfying than smoking combustible cigarettes on account of the lack of throat hit, the delivery of nicotine and the ‘*too light*’ flavour of the tobacco. These more variable experiences contributed to participants discontinuing IQOS or using cigarettes alongside IQOS.

*I got tired of it after a while… I wasn’t getting long-term… satisfaction from it, and I don’t think that my craving for cigarettes actually decreased as much as I would have liked it to… there wasn’t the same fulfilment.* (Hayden, 48, ex-IQOS user, daily smoking)

#### Sensory experiences: sight, smell, taste and touch

The influence of IQOS on the senses of sight and smell regularly explained why participants experimented with IQOS. Participants were attracted to try IQOS due to its ‘*slick*’ and ‘*stylish*’ appearance, discreet size and high-quality finish, which differed to ‘*bulky*’, ‘*huge*’, ‘*ugly*’ tank-style e-cigarettes. The promise that IQOS created little smell also appealed to participants and stood in contrast with the ‘*horrible*’, ‘*disgusting*’ smell of combustible cigarettes and the ‘*weird*’, fruity odours of e-cigarettes.

*(IQOS) is a nice piece of kit, very, very high quality, it looks very nice… It’s inoffensive, it’s not in anybody’s faces like… a great big vape kind of battery… it was very smart, very small, very stylish.* (Neal, 47, current IQOS user, former smoking)

Participants commonly claimed that the overall sensory experience of using IQOS was equivalent to, or better than smoking combustible cigarettes, which accounted for continued use. They praised aspects of the sight (eg, the visual appeal of the branded HEETS packets, the ‘*clearer*’ and reduced volume of emissions, the lack of staining on fingers and teeth, and the cleanliness of no ash), smell (eg, the odourless nature of used HEETS and the lack of residual smell on hands, breath, clothes and furnishings), taste (eg, the comparable taste with combustible cigarettes and the lack of aftertaste) and touch (eg, the tactile feel of the device and the familiar circumference and texture of HEETS in the fingers and on the lips).

*You can use IQOS and not feel bad about yourself afterwards in terms of like the aftertaste, the smell… you just feel nice*. (Yulia, 19, current IQOS user, less than monthly smoking)

Furthermore, participants who had smoked combustible cigarettes after switching to IQOS described how the ‘*dirty*’, ‘*smelly*’ and ‘*disgusting*’ sensory experiences contrasted with ‘*cleaner*’ and ‘*less messy*’ use of IQOS. Consequently, participants often envisaged continuing to use IQOS even if the health-related harms were identified as equivalent to, or worse than smoking combustible cigarettes due to the overall ‘*cleaner*’ experience.

*If there was a headline that says that IQOS was actually more harmful than cigarettes… I probably would still use them… there’s less smell, there’s no ash, it’s cleaner.* (Sanjay, 43, current IQOS user, less than monthly smoking)

Reports of less positive sensory experiences were limited to not liking the taste of HEETS and noticing an unpleasant smell when IQOS started to heat. Indeed, some described that when they inserted HEETS into the holder and turned it on, it smelt ‘*burnt*’ or like ‘*sewage*’, ‘*a fart*’ or ‘*manure*’. These experiences did not deter initial use of IQOS but sometimes discouraged ongoing use.

### Practical factors

#### Accessibility

Until availability of HEETS expanded, early users noted that purchasing HEETS from an IQOS store was inconvenient; sometimes impeding use. Similarly, variable availability and reduced availability outside of London interfered with IQOS use when visiting other cities or when travelling overseas. When faced with issues of availability, participants temporarily discontinued IQOS and smoked combustible cigarettes instead.

*It’s not accessible everywhere yet. So that… leads you to… buying a cigarette packet anyway, because the area that you’re hanging around might not have HEETS.* (Yusuf, 25, current IQOS user, weekly smoking)

Although some praised the availability of HEETS online, they noted that bulk purchasing cartons of HEETS encouraged ongoing IQOS use and deterred stopping.

#### Use in private and public places

Commonly, participants were encouraged to continue using IQOS as they could use it in places where they would not smoke. Prompted by the reduced smell and appearance of emissions, participants used IQOS in their homes and cars. Confident that they would be undetected, they also used IQOS in public places where smoking and vaping were prohibited, including trains, non-office workplaces, pubs, restaurants, the grounds of a psychiatric hospital and at boarding school. While beneficial on the one hand, participants sometimes disliked that they used HEETS more than combustible cigarettes and as a result, they had less incentive to quit.

*I’m totally conscious because I can smoke in more places, I probably do… I certainly have twice the number of HEETS as I used to have cigarettes.* (Raj, 43, current IQOS user, former smoking)

At the same time, participants expressed uncertainty about the rules governing IQOS use in public, relative to smoking and vaping.

#### Maintenance and operation

Participants reported that the need to charge and clean IQOS was burdensome and hindered IQOS use, especially if their device was faulty or broken. Accordingly, they drew parallels between IQOS and e-cigarettes, contrasting them with the simplicity of smoking combustible cigarettes.

*You can’t use the HEETS in any other way. Whereas with a cigarette you just go and ask someone for a cigarette… With the HEETS, if that technology fails, you’re lost without it.* (Sean, 52, current IQOS user, former smoking)

Although having to re-charge the holder in the pocket charger between HEETS prevented participants from ‘*chain-smoking*’ with IQOS, it prompted them to smoke combustible cigarettes when they wanted to chain-smoke.

### Psychological factors

### Ritual and routines

Participants were encouraged to continue to use IQOS as it mirrored their rituals and routines of smoking combustible cigarettes. They drew parallels between the situations when they used IQOS and identified similarities between taking a HEET out of the packet, the hand-to-mouth action of using IQOS, the amount of puffs/time a HEET lasted and the definitive end point of finishing a HEET—all of which they contrasted with using e-cigarettes.

*I like… that the HEETS kind of simulate a cigarette packet… it’s the action of taking out a cigarette, I think behaviourally like it definitely has played a role, because I feel like I’m still smoking. Whereas with a vape I feel like I’m just inhaling some nice flavour.* (Karina, 22, current IQOS user, monthly smoking)

Finally, participants discussed how charging and cleaning IQOS led them to develop new habits and rituals. However, participants were divided on the extent to which these new practices helped or hindered continued use of IQOS.

### Trailblazing and trendsetting

Some participants first used IQOS as they were attracted to the ‘*cool’* design and technological appeal. Indeed, individuals recalled their enjoyment of being an ‘*early adopter*’ or ‘*first mover*’ when few others in the UK knew of or used IQOS. Using IQOS, participants felt ‘*trendy*’, ‘*futuristic*’ and at the forefront of technological developments in smoking—perceptions that were reinforced when others complimented them on IQOS and when they visited ‘*high-end*’, ‘*Apple-like*’ IQOS stores. Accordingly, the ‘*status symbol*’ of IQOS, and the more ‘*exclusive*’ experience it offered, differed from combustible cigarettes and e-cigarettes and inspired participants to pursue IQOS.

*They buy it because it’s cool and looks nice. Honestly no one cares that its less hurtful or whatever, it’s just like the new thing.* (Sofia, 20, ex-IQOS user, daily smoking)

In opposition, other participants shared worries about the newness of IQOS. They raised anxieties that IQOS may not have been sufficiently tested, questioned whether IQOS fit with their image, worried about attracting unwanted attention and feared the reactions of others. Participants explained that these concerns stemmed from their mistrust of PMI and fears of having fallen ‘*victim*’ to their advertising. Although such views led participants to question their use of IQOS, they continued using IQOS.

### Social factors

#### Influence of others

Participants reported that family and friends who used IQOS often promoted it to them as an alternative to smoking and urged them to try it. Other participants experimented with IQOS to appease non-smoking partners, or in response to seeing IQOS users in Japan or mainland Europe.

Nevertheless, once participants had switched to IQOS, they were sometimes tempted to smoke combustible cigarettes when among cigarette smokers.

*I buy them (combustible cigarettes)… when I go party and especially with friends who smoke… It’s more of a social thing… to build the rapport with the person I think it’s easier to follow the lead they are doing, instead of trying to break it.* (Alexei, 27, current IQOS user, monthly smoking)

#### Acceptability

Participants discussed several social consequences of using IQOS which encouraged their ongoing use. First, on account of the reduced visibility, amount, smell and perceived harm of the emissions, participants said that IQOS was ‘*better*’ to use around non-smokers than combustible cigarettes or e-cigarettes.

*[I] feel less guilty smoking the IQOS around other people because… as far as I know it’s not as harmful to secondhand smokers as cigarettes.* (Yasmina, 25, current IQOS user, weekly smoking)

Furthermore, as using IQOS attracted little attention, participants felt more comfortable using it in public or when in the company of non-smokers. Participants felt less ‘*vilified*’ and experienced less stigma and negative judgement with IQOS than when they had smoked or vaped.

*It’s satisfying socially… I don’t have to exclude myself from any conversations… anymore… because when I was smoking, I really felt uncomfortable standing with people who didn’t smoke, because that smoke is quite unpleasant*. (Sean, 52, current IQOS user, former smoking)

Others attributed improvements in relationships with romantic partners and work colleagues to the absence of cigarette smoke on their breath, hair, and clothes.

Meanwhile, participants also criticised IQOS for limiting shared social experiences with other smokers. For example, users did not pass and share HEETS or IQOS in the same way as combustible cigarettes and they missed spontaneous conversations sparked by borrowing a lighter. Furthermore, IQOS users occasionally worried about being judged by cigarette smokers due to perceptions that IQOS was only affordable to the wealthier middle classes. These experiences discouraged them from using IQOS in situations where they did not want to be seen as ‘different’ or where they wanted to bond with cigarette smokers.

*It’s not as social an experience as smoking… because no one uses IQOS and there’s only one, one device… (with cigarettes) you can pass around your packet, say, ‘have one of these’. You can’t do that with IQOS… so it didn’t feel like there was that shared moment that you have when you smoke with friends*. (Peter, 39, ex-IQOS user, daily smoking)

## Discussion

As far as we are aware, this is the first independent qualitative study of IQOS users/ex-users outside of Japan and Switzerland, and the first to use in-depth one-to-one interviews.^28 29^ Similar to previous studies, initiation was encouraged by novelty and the sleek appearance of IQOS.^29^ Participants commonly used IQOS to reduce the harms of smoking, also reflecting earlier findings.^28^ Perceptions of reduced harm relative to smoking were underpinned by messages that IQOS emits 95% less chemicals than cigarettes, and were reinforced by the cleanliness of using IQOS, the lighter sensory perceptions, the simple branding of HEETS packets with a text-only warning and the lack of pictorial health warnings. Optimism helped maintain the belief that IQOS was less harmful than smoking, yet participants understood that IQOS was not risk-free. Visual and sensory similarities between HEETS and combustible cigarettes also encouraged continued use, along with the lack of smoke and lingering smell. Yet, the lack of smell also reduced thoughts about stopping IQOS, in contrast to how the lingering smell of combustible cigarette smoke sometimes served as a reminder to quit.

The high initial start-up cost was a barrier to trying IQOS, while the cheaper price of HEETS compared with combustible cigarettes (but not rolling tobacco or e-cigarettes) encouraged continued use. Perceived physical health improvements contributed to continued use, whereas reduced satisfaction deterred continued use. Practical factors, such as availability of HEETS, and wanting to bond in social interactions with cigarette smokers discouraged some situational use but not overall use.

No single factor above all else was important for IQOS use. For example, despite the importance of reduced harm, participants explained that if IQOS turned out be equally or more harmful than combustible cigarettes, they would likely *still* use IQOS due to other perceived benefits. Furthermore, various factors interacted with one another and influenced use. For example, the ability to use IQOS in more places combined with perceptions of reduced harm led some participants to use more HEETS than combustible cigarettes, despite initial intentions to reduce smoking behaviours. In addition, even though practical challenges such as charging deterred use, the same finding emerged in our early qualitative e-cigarette research.^32^ It is worth noting that e-cigarette users/the market adapted to meet these challenges, and IQOS users/manufacturers will likely do the same.

Many of the influences on IQOS use were similar to those identified in e-cigarette research—harm reduction encouraged use^32^ whereas practical factors discouraged use.^33^ However, unlike previous studies, participants described the reasons that they used IQOS by comparing the benefits of IQOS with the perceived drawbacks of e-cigarettes. Compared with e-cigarettes, IQOS smelt better, was less bulky, felt more like combustible cigarettes in the hands and mouth, and HEETS had an end point.

### Policy and research implications

Our findings on how reduced harm perceptions, the packaging, price and indoor use of IQOS influenced use have important policy and research implications. Research on how tobacco/nicotine policies, such as health warnings and taxation/price, and industry behaviour interact to influence switching from combustible cigarettes to IQOS is needed. The finding that some participants used up to twice as many HEETS as combustible cigarettes warrants follow-up considering a study comparing HTP and combustible cigarette costs^34^ assumed that people smoked equal numbers of combustible cigarettes and heated tobacco sticks. As current smoke-free policies in the UK do not officially cover IQOS, the ability to use IQOS undetected also warrants further research, as does research into the content of IQOS emissions and potential harms to bystanders. Given that the FDA has now authorised IQOS for the US market, our findings may also help inform research into US policy options.

As our study took place in London, our findings may not be generalisable. Yet, many of our findings aligned with studies of IQOS users in Switzerland and Japan,^28 29^ suggesting that they will likely be applicable to other contexts. Given most participants were currently using IQOS, our findings may reflect more positive experiences of use. Although reasons behind individuals’ discontinuation of IQOS was uncovered, additional explanations may exist given our small sample of ex-users. In addition, as most of the sample had negative experiences of e-cigarettes, we cannot speculate how those with more positive experiences of e-cigarette use may experience IQOS. Lastly, although the team regularly discussed the coding process, having a single coder could be criticised for potential bias.

Nevertheless, this is the first independent qualitative interview study of IQOS, which was deliberately informed by consultations with experts and smokers/ex-smokers to prioritise key areas of research and policy relevance, and used multiple recruitment methods to access a diverse sample with a range of views and experiences.

## Conclusion

Overall, this study found that a variety of factors influenced IQOS initiation and continued use. The perceived drawbacks of IQOS and the continuing appeal of combustible cigarettes led some people to stop using IQOS altogether, and others to stop situational use of IQOS, smoking combustible cigarettes instead. Several factors that encouraged/discouraged IQOS use may be considered by regulators if they wish to influence use, including packaging, labelling, risk communication, taxation and smoke-free policies.

**What this paper adds**

**What is already known on this subject**

- Smokers in Japan and Switzerland appear to be taking up IQOS use due to several reasons, including lower perceived harm and cleanliness.

**What important gaps in knowledge exist on this topic**

- Outside of Japan and Switzerland, independent consumer research on IQOS is mostly non-existent, particularly in countries such as the UK where strong tobacco control policies exist and the e-cigarette market is well established.

**What this paper adds**

- UK smokers and ex-smokers endorsed similar reasons for using IQOS as those in Switzerland and Japan, particularly reduced harm, smell and cleanliness. Perceptions of harm reduction were underpinned by industry pronouncements, the non-combustible process involved, and the appearance of IQOS-related packaging and labelling. Some advantages of IQOS over e-cigarettes were reported such as reduced emissions, smell, and clear start and end point of a bout of use.
