# Supplementary material for: Factors that influence smokers’ and ex-smokers’ use of IQOS: a qualitative study of IQOS users and ex-users in the UK
Source: Tob Control. 2020 Jan 15;30(1):16–23. doi: 10.1136/tobaccocontrol-2019-055306 (PMC7803910; doi:10.1136/tobaccocontrol-2019-055306)
Supplement: Supplementary data [file tobaccocontrol-2019-055306supp001.pdf]

Supplementary Table 1. *Daily IQOS users (n=20) – Cigarette smoking and e-cigarette status*

**Cigarette smoking**	**Daily (n = 2)**	**Weekly (n = 3)**	**Monthly (n = 3)**	**Less than monthly (n = 5)**	**Ex-smoker (n = 7)**	**Total (n = 20)**
**E-cigarette use**						
**Daily**	0	0	1	0	0	1
**Weekly**	0	0	0	0	0	0
**Monthly**	0	0	0	0	0	0
**Less than monthly**	1	0	0	0	0	1
**Past use**	1	3	2	4	7	17
**Never**	0	0	0	1	0	1

Supplementary Table 2. *Weekly IQOS users (n=2) – Cigarette smoking and e-cigarette status*

**Cigarette smoking**	**Daily (n = 1)**	**Weekly (n = 1)**	**Monthly (n = 0)**	**Less than monthly (n = 0)**	**Ex-smoker (n = 0)**	**Total (n = 2)**
**E-cigarette use**						
**Daily**	0	0	0	0	0	0
**Weekly**	0	0	0	0	0	0
**Monthly**	0	0	0	0	0	0
**Less than monthly**	0	0	0	0	0	0
**Past use**	1	1	0	0	0	2
**Never**	0	0	0	0	0	0

Supplementary Table 3. *Ex-IQOS users (n=8) – Cigarette smoking and e-cigarette status*

**Cigarette smoking**	**Daily (n = 4)**	**Weekly (n = 0)**	**Monthly (n = 1)**	**Less than monthly (n = 1)**	**Ex- smoker (n = 2)**	**Total (n = 8)**
**E-cigarette use**						
**Daily**	0	0	1	0	0	1
**Weekly**	0	0	0	0	0	0
**Monthly**	1	0	0	0	0	1
**Less than monthly**	0	0	0	0	0	0
**Past use**	2	0	0	1	2	5
**Never**	1	0	0	0	0	1
